# Success Factors for Community Health Workers in Implementing an Integrated Group-Based Child Development Intervention in Rural Bangladesh

**DOI:** 10.3390/ijerph18157891

**Published:** 2021-07-26

**Authors:** Tania Jahir, Peter J. Winch, Elli Leontsini, Sharon T. Hwang, Farzana Yeasmin, Khobair Hossain, Jyoti Bhushan Das, Ruhul Amin, Tarique Md. Nurul Huda, Jesmin Sultana, Rizwana Khan, Fahmida Akter, AKM Shoab, Rezaul Hasan, Helen O. Pitchik, Fahmida Tofail, Lia C. H. Fernald, Stephen P. Luby, Mahbubur Rahman

**Affiliations:** 1 Environmental Interventions Unit, Infectious Diseases Division, International Center for Diarrheal Diseases Research, Bangladesh (icddr,b), Mohakhali, Dhaka 1212, Bangladesh; fyeasmin@icddrb.org (F.Y.); khobairbd@gmail.com (K.H.); jyoti.das@icddrb.org (J.B.D.); lianamin111@gmail.com (R.A.); tarique.huda@icddrb.org (T.M.N.H.); jesmin.sultana@icddrb.org (J.S.); rizwana.khan@icddrb.org (R.K.); fahmida.akter@icddrb.org (F.A.); akmshoab@icddrb.org (A.S.); rezaul.hasan@icddrb.org (R.H.); mahbubr@icddrb.org (M.R.); 2 Department of International Health, John Hopkins Bloomberg School of Public Health, Johns Hopkins University, Baltimore, MD 21205, USA; pwinch@jhu.edu (P.J.W.); eleontsi@jhu.edu (E.L.); sharon.tae.hwang@gmail.com (S.T.H.); 3 Division of Epidemiology, School of Public Health, University of California, Berkeley, CA 94720, USA; hpitchik@berkeley.edu; 4 Nutrition and Clinical Services Division, International Center for Diarrheal Diseases Research, Bangladesh (icddr,b), Dhaka 1212, Bangladesh; ftofail@icddrb.org; 5 Division of Community Health Sciences, School of Public Health, University of California, Berkeley, CA 94720, USA; fernald@berkeley.edu; 6 Division of Infectious Diseases and Geographic Medicine, Stanford University, Stanford, CA 94305, USA; sluby@stanford.edu

**Keywords:** community health workers, integrated intervention, group sessions, early child development, maternal mental health, prevention of lead exposure, Bangladesh

## Abstract

Community Health Workers (CHWs) can effectively implement maternal and child health interventions, but there is paucity of evidence on how to integrate child stimulation into these interventions, and their delivery at scale. In rural Bangladesh, CHWs implemented an intervention integrating psychosocial stimulation, nutrition, maternal mental health, water, sanitation, hygiene (WASH) and lead exposure prevention. In each of 16 intervention villages, one CHW worked with 20 households. CHWs bi-weekly held group meetings or alternated group meetings and home visits with pregnant women and lactating mothers. We assessed the intervention through five focus groups, four interviews and one group discussion with CHWs and their supervisors to explore success factors of implementation. CHWs’ training, one-on-one supervision and introduction by staff to their own community, and adoption of tablet computers as job aids, enabled successful session delivery to convey behavioral recommendations. CHWs reported difficulties delivering session due to the complexity of behavioral recommendations and struggled with age-specific intervention material. Young children’s attendance in group sessions generated distractions that undermined content delivery. We identified ways to minimize the difficulties to strengthen intervention-delivery during implementation, and scale-up. Iterative revisions of similarly integrated interventions based on qualitative evaluation findings could be delivered feasibly by CHWs and allow for implementation at scale.

## 1. Introduction

In low- and middle-income countries (LMICs), millions of children under 5 years old do not reach their full potential for physical and cognitive development [1,2]. Lack of responsive stimulation at home, inadequate nutrition, and infectious diseases in the first 24 months of life, increase the risk for poor cognitive development [3]. Optimal early child development is supported by nurturing care that can be facilitated through a supportive environment, stimulating interaction with caregivers and early learning [4]. Interventions that focus on increasing responsive stimulation in young children under two years of age improve child development outcomes immediately following intervention completion [3]. Two key questions for policymakers and program managers regarding child stimulation interventions are:

(1) Can they be implemented by community health workers (CHWs) at a large scale? and
(2) Can they be integrated with other maternal and child health and nutrition interventions, without losing their effectiveness?

CHW refers to any person, recruited locally, to implement various community-level interventions, whether by government, non-governmental, or faith-based organizations [5]. According to the 2015 National Health Policy of Bangladesh, CHWs are mainly female, and work for both government and non-government organizations. They are mandated to provide a variety of domiciliary and community-based services, including prevention, education, and screening, as well as essential care and commodities and data gathering [6].

Implementation at scale: There are several examples of successful implementation of child stimulation interventions delivered through existing services at smaller scale. In Colombia, a child stimulation intervention was successfully delivered by female community leaders for 18 months, where weekly home visits improved cognitive and receptive language scores of children [7]. In rural Pakistan, a child stimulation intervention was successfully delivered by Lady Health Workers through a combination of home visits and group sessions [8].

The recommended minimum frequency of one-on-one contact between CHWs and beneficiaries is every two weeks [9]. Constraints of personnel and resources make this frequency of contact difficult to achieve in routine public health programs implemented at scale. Including group sessions for child stimulation in routine CHW work may be a feasible way to deliver these interventions at the frequency required.

Integration with other interventions: In resource-constrained settings, CHWs can effectively deliver integrated key maternal and child health interventions [10]. Several factors contribute to CHW performance, including the nature of the task and time needed for delivery, training, equipment, and nature of supervision they receive [11,12,13]. Inadequate training decreases motivation and self-confidence of CHWs [14]. Poor performance of CHWs is influenced by a weak health system, limited resource flow, weak managerial and organizational systems, and barriers to access of services at the community level [14,15].

Effective interventions implemented through CHWs to promote child stimulation recommendations have been either stand-alone interventions or were integrated with only one other component (as opposed to integration with multiple components). Children who received an intervention delivered by Lady Health Workers in Pakistan integrating child stimulation and a nutrition component achieved higher development scores on cognitive, language, and social-emotional domains at 12 months of age compared to those who did not receive this intervention [8]. In a randomized controlled trial in Bangladesh an integrated child stimulation and maternal health intervention in a hospital setting improved child development [15,16].

This study examined CHW enablers and constrains in implementing an intervention integrating child stimulation along with multiple maternal and child health interventions in rural Bangladesh, to inform integration and future scale-up.

## 2. Materials and Methods

### 2.1. Study Context

**Figure 1:**
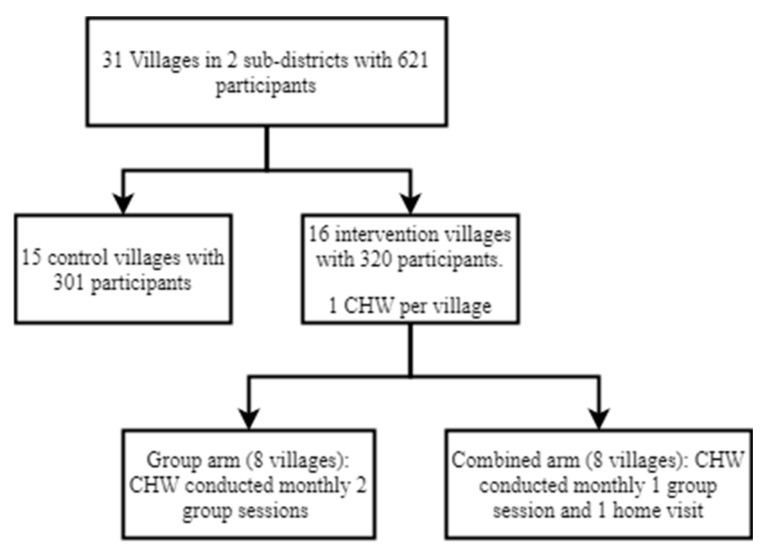
The RINEW study settings and participants.

Our study was nested within a 9-month-long cluster-randomized control trial (ISRCTN16001234), conducted between September 2017 and May 2018, entitled “Research on Integration of Nutrition, Early Childhood Development and WASH (RINEW)”. The trial examined the feasibility and acceptability of implementing these maternal and child health interventions in an integrated manner, to inform future scale-up. The design of the RINEW intervention is described in detail [17] and in brief, this intervention was implemented in 16 villages of two sub districts of Kishoreganj district and enrolled 320 (20 in each village) pregnant women and caregivers of children <15 months of age (Figure 1). We selected this age group because this is the most important time-period of a child’s life to reach their full cognitive development. As the duration of this intervention was 9 months, selecting this age group ensured all enrolled children received the full intervention package before they reach the age of 24 months. The RINEW intervention included traditional maternal and child health components, such as exclusive breastfeeding, complementary feeding, and WASH, integrated with components new to the CHWs including early childhood stimulation, maternal mental health, and prevention of lead exposure (Table 1). The maternal mental health component was an adapted version of the “Thinking Healthy” program aiming to reduce maternal depression in the pre- and postpartum period [18,19]. The nutrition component incorporated behavioral recommendations for maternal and child nutrition from the national nutrition guidelines of the Government of Bangladesh (GoB) and was adapted from the Rang-Din study in Bangladesh [20]. Behavioral recommendations related to WASH were adapted from the WASH Benefits Bangladesh study [21], and behavioral recommendations to reduce lead exposure were developed based on previous formative research [22]. The individual components were integrated and adapted following a systematic approach which included a situation analysis, two months of pilot implementation, and the incorporation of ongoing feedback from participants and the implementers [23].

The RINEW intervention was delivered in two intervention arms (Figure 1), one which consisted of only group sessions, with scalability in mind, and one which consisted of alternating group sessions and home visits to reflect the state of the art [8]. The RINEW trial demonstrated that, compared with the control arm, children in both intervention arms received more stimulating play activities and play materials, and their caregivers had improved knowledge of lead [17]. Additionally, those who received the group-only intervention had improvements in child development indicators in the communication, fine motor, gross motor, and personal social domains, maternal mental health, and more households had a handwashing station with soap and water [17].

### 2.2. Recruitment and Training of CHWs

CHWs were selected based on the following primary criteria: female, less than 38 years of age and had completed at least secondary education (Table 2). Considering the comparatively complex study contents, we recruited women from this age range so that we would be able to train them adequately. An additional consideration is that many women stop serving as CHWs when they start their family. Therefore, this age group is more representative of CHWs currently serving. Finally, we used tablet computers to deliver session contents. In Bangladeshi rural context, older women are less familiar and experienced with such technology. Applicants who passed a written and verbal examination were selected to work as CHWs. CHWs participated in three types of training: basic, tablet application, and refresher [17]. Prior to the intervention activities, they received an eight-day residential training followed by demonstration sessions in the classroom and in real community settings. Trainers and training materials addressed how to build rapport with community residents, communication skills, facilitation skills, the use of study materials including flipcharts, tablet computers, posters, and food cards. Participatory training approaches such as role play, field practice, and quiz sessions with small gifts were included in the training sessions to increase the enthusiasm of CHWs. Expert facilitators were engaged to facilitate the training sessions.

Before the inception of the intervention, the study team introduced CHWs to the community through a community engagement meeting explaining CHWs’ qualifications, types of training they had received, types of activities they would perform, and the households they would visit to deliver the interventions.

### 2.3. Responsibilities and Supervision of CHWs

CHWs worked six days a week, 3–4 h a day and were scheduled to conduct either two group sessions or three home visits in a day. Each CHW received a monthly stipend of 35 USD which was derived from a 20 USD local unskilled laborer’s equivalent earning working 5 days a week [21], adjusted for recent inflation. CHWs were also provided with an experience certificate upon completion of the RINEW study.

In each village the 20 participating mothers were gathered into groups of 4–6 based on geographic proximity. CHW-led group sessions and home visits took place in the courtyard, room, or corridor of a participant’s house. CHWs reminded mothers of upcoming sessions at least one day prior to each session. Tablet computers were used as job aids by CHWs to guide them through age-specific intervention content, and to show videos and images to the participants related to the session contents. CHWs also distributed a series of age-specific low-cost toys and books, nutritional supplements and hygiene-related commodities to the enrolled mothers and their children, described in more detail in Pitchik et al. [17].

Four supervisors were assigned to supervise the 16 CHWs. During each two-week period, each supervisor monitored at least one group session or three individual home visits per CHW. Supervisors observed session delivery by the CHWs, provided feedback and on-the-job training. Their demographic characteristics are summarized in Table 2.

### 2.4. Methods of Qualitative Evaluation

We conducted a qualitative evaluation at two different time points: (1) 4 months after the intervention started, in December 2017 and (2) after 9 months of intervention delivery, when the intervention was complete, in May 2018, to examine CHW success factors and constraints of implementing the RINEW intervention. All 16 RINEW CHWs and their four supervisors were interviewed during each time point. Over the two time points the study team conducted five focus groups with CHWs, four in-depth interviews with the four supervisors (one with each) and a final discussion with the four supervisors together, at the end of the RINEW intervention (Table 3). Each CHW participated in a focus group twice, once per time point. Experienced and well-trained qualitative researchers conducted all data collection in Bengali, the participants’ native language. Field guides contained probes on enablers and difficulties of CHW training, delivery and management of group sessions, home visits, and intervention content, probing sequentially for all the individual intervention components. All data collection was audio recorded. Notes were also taken during the focus groups and the group discussion on unspoken but important responses. On average, interviews lasted for 45 min and focus groups and the final group discussion lasted for 90 min. Focus groups and final discussion were conducted by two researchers at a time: one as a facilitator, and other as notetaker and co-facilitator.

All recorded data were transcribed in Bengali. The research team generated deductive codes prior to the data collection according to the topics on the field guides, including enablers and difficulties of CHW training, delivery and management of group sessions, home visits, and intervention content. Transcripts were coded by four coders using ATLAS.ti version 7.5.10 (ATLAS.ti Scientific Software Development GmbH, Lietzenburger Str. 75, D-10719, Berlin, Germany). While coding, new emerging (inductive) codes were also included. There was high agreement among coders, because the study themes were more observable and directly related to project activities. Whenever there was a coding disagreement, the coder brought it up for discussion among all coders and agreement was reached by consensus. Coded textual data from focus groups and interviews were then summarized together in English by thematic content. Member checking of focus group and in-depth interview findings was conducted during the final discussion with the four supervisors. Direct quotes from the transcripts were selected during data analysis to illustrate each theme.

### 2.5. Ethical Consideration

Prior to the data collection, we obtained written informed consent from all participants. The study protocol was reviewed and approved by the Institutional Review Board (IRB) of icddr,b in the year 2016 (Protocol no: PR#16037) and the University of California Davis in 2017 (approval no. 968287-2).

## 3. Results

First, we present factors that were identified by CHWs and their supervisors as positively influencing implementation activities. Second, we present some difficulties CHWs faced during training and delivery of the RINEW intervention.

### 3.1. Enablers of Successful Intervention Implementation

Most CHWs identified receiving one-on-one supervision, being introduced by project staff during community engagement meetings, receiving good quality training, delivering the intervention in their own community, delivering the child stimulation component of the intervention, and the delivery of sessions with tablet computers as facilitators of session delivery. CHWs mentioned that the quality of the training sessions, and quick and easy communication with their supervisors gave them confidence to carry out their job responsibilities. A CHW aged 32 with previous work experience stated the following during a focus group:

*The behavior and attitude of all our supervisors and other officials was very positive and nice to us. This also raised our acceptance and respect in the community. In my previous job, I did not find this. This is another fact, I would love to join another project of iccdr,b, if I get a chance.*

CHWs reported that during training sessions they could grasp the intervention content through in-house discussion and field tests. They also mentioned that the training facilitators were good at delivering training contents and explained everything using easy language and appropriate examples.

Being from the same community allowed them to build rapport easily with participants. They felt that they could communicate with mothers at any time, and this facilitated the formation of close bonds with the community.

One CHW, age 30, who had completed secondary education, explained:

*I could enter my assigned households at any time; even if mothers were absent, I experienced a warm welcome from their family members. Sometimes I waited alone in their room; they had trust upon me and allowed me to sit in their own room in absence of the mother.*

CHWs identified that delivery of the child stimulation component allowed them to build and maintain good rapport with the enrolled families, which was beneficial for the delivery of the other intervention components as well, as mothers enjoyed activities such as playing with toys, reciting rhymes, and learning with colorful picture books with their children. These types of activities need close engagement with mothers and their children, and they brought CHWs and enrolled participants together. Learning to interact with children in their early life also benefitted the mothers, making for enjoyable sessions.

In an in-depth interview, a female supervisor recalled:

*While observing sessions I found, mothers become very happy when the CHW showed them how to play with toys with their babies and they could do it properly. Mothers were interested and very proud when they found their babies could properly follow CHW’s instruction.*

Furthermore, delivering sessions with a tablet computer was an attraction for both CHWs and mothers. CHWs did not need to remember all the relevant information as all content was uploaded in the tablet computer application. It enhanced their level of confidence while talking to mothers in the group sessions, and it was an object that signaled importance to community members.

One CHW, aged 32, said:

*As we have a tablet computer in our hand, people respect us because they thought we are doing a very high-class job.*

### 3.2. Difficulties of Implementing the Integrated Intervention

CHWs and their supervisors described several hurdles the CHWs faced while implementing the RINEW intervention, summarized in Table 4. Some noteworthy points are discussed in the sub-sections below.

#### 3.2.1. Problems Faced Attending the Required Training

Most CHWs reported difficulty consistently paying attention to content during residential training which had to be lengthy due to the multiple intervention components and their age-specific variation. This happened because they had to leave their family members at home and stay away from home for more than a week. Rural Bangladeshi women usually perform all household chores including cooking, serving, feeding, and cleaning and they rarely spend time outside their home. One CHW, aged 28, stated:

*I never stayed overnight outside without my family members. Yes, sometimes I stayed at my parents’ house with my children, but after my marriage, I did not stay outside alone. I always think of what my children are doing and what my in-laws would think about me. They must be thinking that I’m staying there so that I might not do household chores. I always remain concerned whether any of my family members is calling me over phone. That’s why I could not pay full attention to the sessions.*

Some CHWs (4) stated problems they faced to attend the daily and day-long training sessions as the venue was located in a central point of the study area and far away from their homes. They reported problems attending sessions on time and getting back home before dusk.

One CHW, aged 32 years, with previous work experience of 2–3 years, reported:

*It takes two hours to come to the training venue from my residence. I started very early in the morning. For this reason, I needed to wake up early and complete all household chores. I found my entire attempt to join training session on time, were in vain, because I needed to wait for vehicle. Sometimes it took an hour to get into a bus to reach the training venue.*

Another CHW, aged 38, shared:

*Sometimes, after completing training sessions, when I get back to my home, it is almost night. My in-laws are not happy with that. They did not allow me to stay outside till night. Sometimes my brother-in-law says harsh words to me due to my late arrival at home.*

#### 3.2.2. Difficulties in Conducting and Managing Group Sessions

Young children’s presence was needed in the sessions for the CHWs to demonstrate child stimulation activities, and coach caregivers to practice them, but problems that arose from having children attend sessions were reported by all 16 CHWs. On average, six children were present in a typical session and were up to 24 months of age. A typical scenario faced in a group session is depicted in Box 1. This is how one CHW expressed her difficulties with interacting with children of different ages:

*We were supposed to show the baby how to play with toys according to their age group in one session, I was showing a baby how to play with a flower puzzle, then babies from other age groups also demanded the same toy. At the time, I did not have enough flower puzzles. The children continued to cry and grabbed the toy from other children. Some mothers left early as the sessions became chaotic and loud. As a result, I could not complete that particular session.*

Box 1 **Description of a child stimulation activity.**

This is a small village named “Manikkhali”. A sunny and very hot day of April. Six mothers; 2 pregnant and 4 lactating (with 3-, 6-, 13- and 19-month-old children) gathered in a courtyard. Now it is 10:30 am and all mothers are sitting “U” shape on a mat. A CHW named Rohima is leading the group through a child stimulation exercise meant to promote a child’s language and motor development.

She then takes one toy out of the bag and shows the mothers how to play with it and what the advantages are. After demonstrating, she tells mothers to sit face-to-face with their children. She places a toy in front of the mother–baby duo and tells the mother to play with baby. Meanwhile, another baby tried to grab the toy. Then two children start fighting over the toy. One mother said, “My boy does not like doll, he likes cars.” Another mother said, “I have two children, if you give one toy, my other baby will not let him play.” At the same time, one baby defecated on the mat. Her mother takes her away to clean. Rohima pauses and tries to console the babies who are fighting for the toy. Already half an hour passed. During this whole situation, pregnant mothers were sitting idly and talking to each other. Rohima tried to attract their attention by saying, “apa look how they are playing, it will help when you deliver your babies.” Rohima manages to reduce the chaos and starts showing a video of breast-feeding positioning and attachment. Some mothers are trying to watch the video, but the children want to grab the tablet. Somehow the video stopped. Rohima is trying to play the video again. Pregnant mothers were not feeling comfortable sitting in one place, one was yawning, and it seems like she is sleepy. Now it is 11:10 am, and Rohima is talking about benefits of handwashing by showing some images in the tablet. Meanwhile, a pregnant mother says, “I’m not feeling well, I want to go to home, and I need to sleep.” Another mother says, “My baby got a scratch on her face by other baby, if his grandparents came to know about it, they won’t allow me to come here for the next session.” Rohima tries to explain to them the importance of being in the session, but they both left. She continues the session with the other mothers; however, in the meantime they lost their patience and interest to the session. Rohima finished the session in a hurry.

Mothers interrupted sessions to care for their babies when they urinated, defecated, or cried, subsequently leading to mothers missing the start, or part of an activity, resulting in difficulties understanding session content, and ultimately losing interest. Pregnant women who participated in the same session felt bored and reported a condition they referred to using the Bengali terms, sharirik-lokkhon or poriborton (physiological symptoms or change). Symptoms included vomiting, drowsiness, dizziness, headaches, stomachaches, cramps, and generalized swelling, all related to sitting idly for extended periods of time. For these reasons, they could not pay full attention to the sessions. One CHW reported during a focus group:

*Pregnant mothers have various “sharirik-lokkhon” if they sit in one place, they feel sleepy and sometimes pain in their body parts. Then we suggested them to stand up and take a walk around. At that time either we paused the session or talked to the lactating mothers.*

#### 3.2.3. Difficulties with Distribution of Equipment and Commodities

CHWs experienced declining community acceptance during the latter part of the intervention period, when some index families were identified to receive additional commodities free of cost, such as handwashing stations and an improved latrine based on the low mid-upper arm circumference (MUAC) of their child, an indicator of a child’s risk for poor health and development. Some participants who had not received these commodities subsequently refused to attend sessions, and during home visits, CHWs were denied entry by these participants and their family members, due to this uneven distribution. They were being blamed for what was perceived as unfair distribution of equipment and commodities.

#### 3.2.4. Problems Faced in Delivery of Session Content

During focus groups, most CHWs reported facing obstacles in delivering the contents from multiple components in a single session. Each intervention session drew from different modules designed to promote behavioral recommendations specific to children’s ages, and CHWs sometimes struggled to give the different modules appropriate weight. The RINEW study was designed to deliver the child stimulation component at the beginning of each session followed by other components (Table 1). The child stimulation content consisted of several fun activities that took up considerable time, after which mothers often lost patience, and CHWs were pressured to finish sessions quickly. Thus, sometimes they were not able to spend enough time explaining the other intervention components.

CHWs faced difficulties achieving trust from the mothers regarding some specific issues. As a part of the nutrition component, CHWs promoted exclusive breastfeeding during the first six months of age. However, mothers fed formula milk to their babies who were <6 months if their doctors recommended it, because they found doctors to be a more credible source of information. Whenever CHWs tried to highlight the adverse impact of failing to practice exclusive breastfeeding, some mother opposed them, referring to their doctors’ recommendations.

Supervisors and CHWs reported problems delivering some technical content, specifically for maternal mental health. In total, 11 out of 16 CHWs and all four of their supervisors reported that this component was the most complex to implement. The adapted “Thinking Healthy” component drew from a variety of concepts related to the mothers’ personal health habits, mothers’ relationship with their children, and mothers’ relationship with family members and neighbors [23]. However, many CHWs struggled to understand this component and felt they had failed to effectively convey it to mothers. Many CHWs were not able to create a supportive environment during group meetings for mothers to share their symptoms and problems related to mental health. One CHW, aged 28, shared that:

*We are telling mothers to share their problem in meetings. Mothers thought if they had any issue with their husbands or in-laws and they share it in group meetings, other mothers of these meetings will taunt them and laugh at them, which will increase their problems rather reducing them.*

As each session consisted of at least three different components, CHWs needed to carry many supplies and communication materials to the session venue. Though the tablet computers reduced the weight as compared to carrying paper versions of some elements, they still needed to carry a large volume of picture books, toys, and nutritional supplements for the children and for mothers to distribute. To illustrate sources of lead exposure, they had to carry a large rusty lead-soldered tin container (where stored puffed rice may absorb lead from lead solder used to repair older containers) as well as polished and unpolished raw turmeric pieces (that might be colored with lead chromate) [22]. It was difficult for them to carry all these materials, especially in the rainy season and along muddy village roads.

## 4. Discussion

The current study identified noteworthy enablers and specific problems implementing a multi-component intervention integrated with child stimulation. These determinants informed several mitigation strategies to overcome the identified difficulties in this study. In addition, they are useful in informing future efforts of scale-up.

There is mounting evidence that child stimulation interventions can be delivered in community settings through various channels [24,25]. In those studies, the child stimulation intervention was either implemented as a stand-alone or integrated with one other component and was delivered to pregnant women or lactating mothers in separate sessions. The RINEW study differed from other recent studies in Bangladesh [16,26] because child stimulation activities were: (a) part of an integrated package that included multiple components including WASH, maternal and child nutrition, maternal mental health, and prevention of lead exposure, and (b) delivered to both pregnant women and mothers of young children <2 years of age in the same group sessions. Moreover, children attending sessions were from a range of ages. Our study was successful in demonstrating a series of CHW enablers and difficulties in delivering such a complex multi-component integrated child development and maternal health intervention in a group format. Conducting assessments at two different time points and maintaining an open dialog with the CHWs allowed researchers and implementers to confirm and continue to support the enabling factors, as well as collaborate and adjust the intervention delivery format so that the identified challenges could be addressed in a timely way. Ultimately, this process generated an intervention that improved child development indicators, stimulation in the home, maternal mental health, and knowledge of lead [17].

Implementing intervention content for child stimulation based on the child’s age group in addition to the WASH, lead, and nutrition components, posed a continued time management challenge for CHWs. To mitigate this, we reduced the number of mothers in a group. Initially, it was 8–10 mothers; later, we reduced this number to 3–6 mothers per group. To scale up the integrated intervention with a larger group, our suggestion is to deliver the child stimulation component for not more than one age group in a single session.

Other successful changes were also made during the intervention following the first qualitative assessment. Management of the materials and supplies required to deliver the group sessions was improved by making lower-weight and less bulky alternatives for some materials to be shown to the mothers, such as replacing the big rusty tin with a replicated small one.

CHWs had an opportunity to use tablet computer technology to deliver session content. It helped them recall complex session contents including age-specific behavioral recommendations and distribute equipment and supplies to session participants. These findings are similar to those from a maternal, neonatal and child health program in India, where use of mobile applications aided the CHWs in remembering important topics, properly distributing time in each session and engaging mothers to the end [27]. Similarly, a systematic review found that tablet computers and other mobile technologies improved the range and quality of services provided by CHWs and can contribute to positive program outcomes [28].

CHWs indicated that they improved their mastery of the content of the entire integrated package through multiple field tests in addition to residential training sessions. This enhanced CHWs’ confidence, they said, to make themselves heard in front of a group and successfully manage 3–6 mothers with their children.

In many contexts, including rural Bangladesh, there are cultural norms against sharing personal information in public and especially with and in front of in-laws [29,30]. Regarding the “Thinking Healthy” component, in response to the CHWs’ difficulties faced due to these cultural norms, we guided them to teach mothers indirectly, by discussing examples of problems faced by other mothers rather than divulging their own problems. The study team introduced storytelling as a way to deliver intervention content [31], and through a comparative story of two fictitious mothers, CHWs explained how affirmative thoughts have a positive impact on health and encouraged mothers to react to their own negative thoughts through reacting to the negative thoughts of the fictitious mother.

In the RINEW study we aimed to recruit CHWs with at least 10 years of schooling. The profiles of CHWs generally recruited by NGOs vary widely, ranging from paid full-time workers such as the cadre of “Shasthya-shebika” of the Bangladeshi NGO BRAC, part-time community health workers (CHWs) as in the WASH-Benefits Bangladesh study [13,32] to NGO-supported community skilled birth attendants (CSBAs), and depot holders and volunteers who receive no monetary benefits, such as community volunteers of the MaMoni project (Bangladesh national strategy for community health workers) [13,21]. Although all CHWs in our study were required to have a secondary education, but many still struggled to fully understand some of the more abstract and complex ideas. For example, even though we used an adapted and easier version of “Thinking Healthy” [19], and introduced storytelling to deliver its content, CHWs still were frequently unable to deliver it effectively. Appropriate training can improve the CHWs’ ability to successfully implement an integrated intervention [33]. To ensure sufficient training on each component the study team arranged nine different refresher training sessions for CHWs and each of the sessions took 2–3 days to complete.

One important intervention recommendation was to continue exclusive breastfeeding until their baby was six months of age [23]. However, mothers were found to follow formal and informal sector health care practitioners’ recommendations regarding formula feeding instead. In the community, even informal sector providers such as unlicensed ‘village doctors’ (gram daktar) may have a higher status than CHWs. A recent study in Peru [34] had similar findings, and in addition to trusting doctors and other’s advice to use formula, mothers reported receiving infant formula free of cost or at a discounted price from doctors and other health care providers. Producers and manufacturers of infant formula targeted doctors and health providers to influence mothers’ decision about child feeding [34]. Further efforts to reduce the influence of companies selling infant formula and to promote exclusive breast-feeding are needed. Efforts to improve the voice and bolster the credibility of CHWs in the eyes of community members could improve their effectiveness compared with sources of information that promulgate harmful messages.

To deliver any community-based health related intervention strong connections to the community are important [35]. Such connections create a sense of ownership over the program and can foster its success [36]. The RINEW study used MUAC as an indicator to select children facing greater disadvantages for additional material inputs. When targeting interventions, based on a child’s MUAC, CHWs reported that they lost the trust of many community members due to the disparity in items provided to different households in the community. We thought that identifying children to receive additional commodities based on their MUAC would have been acceptable as it is an objective assessment. Other studies did not face any problems distributing different types of nutrition supplements based on MUAC measurement [20]. Perhaps, to the community, handwashing stations and sanitary latrines were more visible and expensive, as well as of no apparent relationship to a child’s MUAC, than different types of nutrition supplements. It would be helpful to consider alternatives for targeting based on a child’s MUAC as many of the participants in our sample of women in rural Bangladesh did not understand the rationale underlying this form of targeting. On the other hand, targeting is important to ensure that those with the greatest need have access to limited commodities and other inputs. Based on a previous voucher scheme study that successfully increased women’s participation by targeting only the poorest women in a Bangladeshi community, direct targeting based on socio-economic status instead of MUAC might prove more acceptable [37].

## 5. Conclusions

A distinctive feature of the RINEW study was that child stimulation activities were part of an integrated package that included multiple components and were delivered to both pregnant women and mothers of young children <2 years of age in the same group sessions, in the presence of children from a range of ages. The diversity of the intervention contents and the varying ages of the children posed unique operational and logistical difficulties. The RINEW team addressed these difficulties through a systematic qualitative evaluation process that allowed for feedback, mentoring, and revision of the intervention package and its delivery. Iterative revisions of similarly integrated interventions based on qualitative assessment findings could be delivered feasibly by CHWs or similar community-based providers. This process may also allow for future implementation of integrated child development interventions at scale.

## Figures and Tables

**Table 1:** Session content delivered by CHWs through group and combined arm.

Group Arm (Group + Group)		Combined Arm (Group + Home Visit)	
Session No.	Components Provided	Session No.	Components Provided
1	Introduction to child stimulation, WASH, and maternal and child nutrition	1 (group)	Introduction to child stimulation, WASH, and maternal and child nutrition
2	Child stimulation, and maternal and child nutrition	2 (home)	Child stimulation, and maternal and child nutrition
3	Child stimulation, WASH, and maternal and child nutrition	3 (group)	Child stimulation, and maternal and child nutrition
4	Child stimulation, maternal mental health, and maternal and child nutrition	4 (home)	Child stimulation, maternal mental health, and maternal and child nutrition
5	Lead, child stimulation, and maternal and child nutrition	5 (group)	Lead, child stimulation, and WASH
6	Child stimulation, maternal mental health, and maternal and child nutrition	6 (home)	Child stimulation, maternal mental health, and maternal and child nutrition
7	Child stimulation, and maternal and child nutrition	7 (group)	Child stimulation, and maternal and child nutrition
8	Child stimulation, WASH, and maternal mental health	8 (home)	Child stimulation, maternal mental health, and maternal and child nutrition
9	Child stimulation and WASH	9 (group)	Child stimulation, WASH, and maternal and child nutrition
10	Child stimulation, maternal mental health, and maternal and child nutrition	10 (home)	Child stimulation, maternal mental health, and maternal and child nutrition
11	Child stimulation and WASH	11 (group)	Child stimulation and WASH
12	Child stimulation, maternal mental health, and maternal and child nutrition	12 (home)	Child stimulation, WASH, and maternal mental health
13	Child stimulation and WASH	13 (group)	Child stimulation
14	Child stimulation, WASH, maternal mental health, and maternal and child nutrition	14 (home)	Child stimulation, maternal mental health, and maternal and child nutrition
15	Child stimulation, WASH, and maternal and child nutrition	15 (group)	Child stimulation and WASH
16	Child stimulation, WASH, and maternal and child nutrition	16 (home)	Child stimulation, maternal mental health, and maternal and child nutrition
17	Child stimulation, and maternal and child nutrition	17 (group)	Child stimulation and maternal mental health
18	Child stimulation and summary on WASH, maternal mental health, and maternal and child nutrition	18 (home)	Child stimulation and maternal and child nutrition

**Table 2:** Demographic characteristics of CHWs and their supervisors.

	Group Arm No. of CHW *N* = 8	Mixed Arm No. of CHW *N* = 8	Total No. of CHW *N* = 16	Total No. of Supervisor *N* = 4
Age in years				
20–25	5	3	8	
31–35	3	4	7	2
36–40	0	1	1	2
Education				
Higher secondary	4	4	8	
Secondary	4	4	8	
Postgraduate				4
Religion				
Muslim	7	8	15	2
Hindu	1	0	1	
Marital status				
Married	6	7	13	4
Unmarried	2	1	3	
Monthly family income (in USD) *				
<125	1	5	6	
126–185	4	2	6	
186–250	2	1	3	
>250	1	0	1	4

* 2017 conversion rate used.

**Table 3:** Participants of qualitative assessments.

**1st Qualitative Assessment**			
**Type of Participant**	**Focus Groups**	**Individual Interviews**	**Group Discussion**
CHW	2 (1 with 8 CHWs of group arm, 1 with 8 CHWs of combined arm)	N/A	N/A
Supervisors of CHWs	N/A	4 (1 with each supervisor)	N/A
**2nd Qualitative Assessment**			
CHW	3 (1 with 8 CHWs of group arm, 1 with 8 CHWs of combined arm and 1 with good and poorly performed CHWs)	N/A	N/A
Supervisors of CHWs	N/A	N/A	1 (with all 4)

**Table 4:** Summary of difficulties to implement the integrated child development intervention identified by community health workers and their supervisors.

Type of Barriers	Description of the Barrier
Barriers related to attending training sessions	CHWs faced problems to arrive at training sessions on time due to distance and lack of vehicle. They also could not give proper attention during the lengthy in-house training sessions required, as they felt distracted and concerned about their prolonged absence from their families at home.
Difficulties related to session management	CHWs faced problems as they had to use different materials for different components in a single session and they failed to attract the mother’s attention due to noise.
Barriers related to achieving trust from the community	CHWs faced problems to achieve trust from the community due to targeting mothers for additional commodities depending on mid-upper arm circumference (MUAC) measurement and for “exclusive breastfeeding” mothers trusted doctors and other health professionals more than CHWs.

## Data Availability

The data that support the findings of this study are not publicly available because we did not ask participants to consent to raw data sharing outside of the research team. Public sharing of the data could compromise anonymity and research participants consent.
